# Exploring Volatile General Anesthetic Binding to a Closed Membrane-Bound Bacterial Voltage-Gated Sodium Channel via Computation

**DOI:** 10.1371/journal.pcbi.1003090

**Published:** 2013-06-13

**Authors:** S. G. Raju, Annika F. Barber, David N. LeBard, Michael L. Klein, Vincenzo Carnevale

**Affiliations:** 1 Institute for Computational Molecular Science, College of Science and Technology, Temple University, Philadelphia, Pennsylvania, United States of America; 2 Department of Neuroscience, Thomas Jefferson University, Philadelphia, Pennsylvania, United States of America; 3 Department of Chemistry, Yeshiva University, New York, New York, United States of America; University of Illinois, United States of America

## Abstract

Despite the clinical ubiquity of anesthesia, the molecular basis of anesthetic action is poorly understood. Amongst the many molecular targets proposed to contribute to anesthetic effects, the voltage gated sodium channels (VGSCs) should also be considered relevant, as they have been shown to be sensitive to all general anesthetics tested thus far. However, binding sites for VGSCs have not been identified. Moreover, the mechanism of inhibition is still largely unknown. The recently reported atomic structures of several members of the bacterial VGSC family offer the opportunity to shed light on the mechanism of action of anesthetics on these important ion channels. To this end, we have performed a molecular dynamics “flooding” simulation on a membrane-bound structural model of the archetypal bacterial VGSC, NaChBac in a closed pore conformation. This computation allowed us to identify binding sites and access pathways for the commonly used volatile general anesthetic, isoflurane. Three sites have been characterized with binding affinities in a physiologically relevant range. Interestingly, one of the most favorable sites is in the pore of the channel, suggesting that the binding sites of local and general anesthetics may overlap. Surprisingly, even though the activation gate of the channel is closed, and therefore the pore and the aqueous compartment at the intracellular side are disconnected, we observe binding of isoflurane in the central cavity. Several sampled association and dissociation events in the central cavity provide consistent support to the hypothesis that the “fenestrations” present in the membrane-embedded region of the channel act as the long-hypothesized hydrophobic drug access pathway.

## Author Summary

The molecular mechanisms mediating the pharmacologically induced state of general anesthesia are, in general, poorly understood. Modulation of voltage gated sodium channels is thought to play a major role in anesthesia, as several members of this class of channels show a significant response to general anesthetics. However, the detailed mechanism of inhibition or potentiation of these channels is completely unknown. Recently, the structures of several members of the bacterial family became available, thereby offering the opportunity to shed light on some of these issues. We have performed molecular dynamics simulations on one of these bacterial voltage gated sodium channels, NaChBac, to identify binding sites and access pathways for the volatile general anesthetic isoflurane. We found that isoflurane, at physiologically relevant concentrations, binds the channel at three distinct sites. One site is in the pore of the channel, suggesting that isoflurane may hinder the permeant sodium ions. Surprisingly, we found that this binding site is accessible to the drug even when the pore and the aqueous compartment at the intracellular side are disconnected. In our simulations, the “fenestrations” present in the membrane-embedded region of the channel act as the long-hypothesized hydrophobic drug access pathway.

## Introduction

Voltage gated sodium channels (VGSCs), which mediate the upstroke of the action potential in most excitable tissues, are key targets of anesthetics. The binding site and molecular mechanism of action for local anesthetics have been well characterized in the last few decades, while the role of VGSCs in general anesthetic action is less well understood and both mechanisms continue to be studied. Thus far, the VGSC binding sites for general anesthetics have not been identified. Identifying binding sites and access pathways for volatile general anesthetics is key to understanding their mechanism of action and to designing new drugs.

Sodium channels can be inhibited by a number of compounds, including toxins, quaternary ammonium compounds and local anesthetics [1]. When the pore is open, local anesthetics can enter from the intracellular side blocking conduction of ions. More recent work shows that general anesthetics also inhibit sodium channels, possibly through pore-blocking mechanisms [2]–[7]. Anesthetic block by both local and general anesthetics is preserved in the archetypal bacterial voltage-gated channel NaChBac [4], [8]. Early observations [9] pointed to a local anesthetic binding site inside the channel, and indeed, in Nav 1.2, the binding site suggested by mutagenesis is in the central cavity [10]. The effect of local anesthetics on NaChBac is consistent with a cavity-binding site, although the precise binding pocket is probably not the same as in Nav 1.2 [8]. It is thus conceivable that local and inhaled general anesthetic sites in the channel cavity in NaChBac overlap.

The classical studies of charged local anesthetics and their analogs showed that blocking and unblocking seemed to require open channels. This led to the description of the “hydrophilic pathway” for drug access. However, additional experiments showed that hydrophobic local anesthetics could bind and unbind even when channels are closed [11]–[13]. This finding suggested an additional “hydrophobic pathway” that circumvents the closed gate. However, no structural correlate for this pathway has yet been conclusively identified.

**Figure 1:**
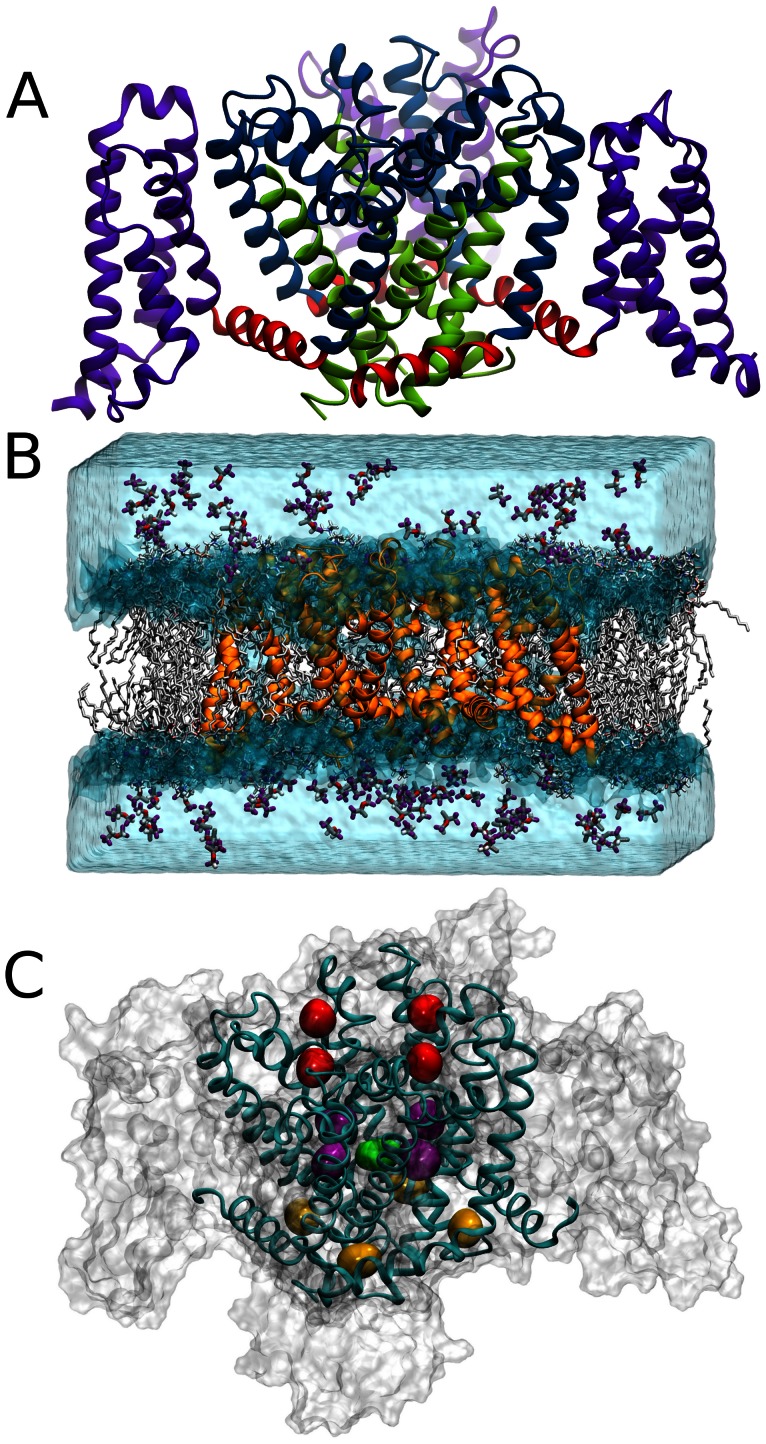
Simulation setup and isoflurane binding sites. (A) Structural domains of NaChBac showing the VSD (purple), the S4–S5 linkers (red) and the pore domain (blue/green) with the S6 helices highlighted in green. (B) Isoflurane flooding simulation system initial setup showing the NaChBac protein (orange) in the POPC bilayer (white) with isoflurane molecules in the aqueous phase. (C) The three binding sites identified by clustering analysis: extracellular site (red), linker site (yellow) and cavity site (purple/green).

Although large mammalian VGSCs have remained resistant to structural characterization, the discovery of the smaller bacterial VGSCs has provided a tool to characterize the structural features of these important channels [14], [15]. In general, the major structural domains resemble those found in the crystal structures of voltage-gated K^+^ channels (Figure 1A). Transmembrane domains S1–S4 form the voltage-sensing domain (VSD), which is connected to the pore domain through the S4–S5 linker. The pore domain, formed by domains S5 and S6 includes the pore-loops (P-loops), which form the ion selectivity filter and may also constitute an inactivation gate [16], [17]. On the intracellular side, four S6-domains form the bundle crossing that constitutes the activation gate [18]–[20]. The S4–S5 linkers form a cuff around the bundle crossing, such that in the presence of a depolarizing stimulus, conformational changes in the VSD widen this cuff, allowing the bundle crossing to open to permit conduction.

A striking feature of the bacterial VGSC crystallized thus far is the presence of the so-called fenestrations [21]–[23]. These fenestrations provide a hydrophobic tunnel through the lipid-embedded portion of the channel to the central cavity, where the consensus local anesthetic site is located. In X-ray crystal structures, lipid tails occupy the fenestrations. These fenestrations may provide the hydrophobic drug access pathway that has been postulated for sodium channels since the 1970s [12], [13], [24].

The available atomic structure of a bacterial VGSC offers the first opportunity to address some of the most fundamental questions about the mechanism of volatile anesthetic action on VGSCs, namely what are the likely structural determinants of general anesthetic effects on VGSCs and could the fenestrations really provide access to the central cavity for small hydrophobic drugs? To investigate these questions, we present the results of a molecular dynamics (MD) simulation study of the bacterial VGSC NaChBac embedded in a lipid bilayer in presence of the inhaled general anesthetic isoflurane. Our unbiased “flooding” technique [25] is a way to flexibly dock isoflurane molecules to high affinity sites of the membrane-bound protein, as well as to thereby suggest energetically favorable drug binding pathways.

## Results

### Identification of isoflurane binding sites

To identify putative isoflurane binding sites, we performed MD “flooding” simulations [25] on a structural model of NaChBac [26] inserted in a hydrated lipid bilayer (Figure 1, A and B, Movie S1). We considered a system comprising the membrane-bound channel along with a large number of drug molecules, initially located in the aqueous phase (Figure 1B). As expected from the Meyer-Overton rule, we observed almost complete partitioning into the membrane within the first 100 ns of MD simulation (Figure S1). In the subsequent MD trajectory, we observed binding to several hydrophobic pockets on the protein surface with at most a single isoflurane molecule in the aqueous phase. Importantly, the conformation of NaChBac was not significantly affected on the time-scale of our MD simulation by the binding of isoflurane molecules, as inferred from the RMSD of the channel along the MD trajectory (Figure S2). The potentially large number of binding sites poses a challenge in identifying which of these may be pharmacologically relevant. To overcome this challenge we applied a two-fold strategy in which we first explored all the possible binding modes of isoflurane, and then used an established data-mining approach [27] to extract distinct configurations from our large data-set of molecular configurations. Specifically, we perform a cluster analysis on the positions of the centers of mass of the whole ensemble of isoflurane molecules and ranked the clustering solutions according to their local density. The rationale for this choice is to prioritize those regions that show continuous occupancy, which would suggest tighter binding of the drug molecule. This analysis revealed three major binding regions (Figure 1C): 1) a region near the selectivity filter, termed the extracellular site, 2) a region near the S4–S5 linker, termed the linker site, and 3) a region within the cavity, termed the cavity site.

Due to the protein's four-fold symmetry, a functional channel has four equivalent copies of each of the three putative binding sites. To investigate whether the three putative sites are occupied symmetrically in the tetramer, and to determine which amino acids line each site, we analyzed the interactions between isoflurane and all the residues within the sites in each subunit. In particular, we monitored the contacts between any atom of the drug molecule and any atom of a given amino acid for each instantaneous configuration after isoflurane partitioning has reached equilibrium. Residues were classified as non-interacting, “possibly” interacting, and “likely” interacting according to the number of configurations in which the contact was detected. For the extracellular site, all amino acids in all four subunits are in contact with the drug. In the cavity site as well as through the fenestrations, most residues are possibly or likely interacting in all four subunits. Intriguingly, the linker site is asymmetrically occupied in two adjacent sites.

### Characterization of isoflurane sites

We used the clustering data and proximity time analyses, in combination with analysis of hydrogen-bonding-like interactions and mobility of the drug molecule in the sites, to structurally characterize the sites and elucidate the key determinants of binding. Furthermore, to assess the pharmacological relevance of these sites, we estimated the binding free energy of isoflurane for each site using well-established FEP methods [28], allowing us to rank the relative free energy at each site. We also estimated binding affinities (Kd) and found that they were within a reasonable physiological range (see Methods) [29].

#### Extracellular site

**Figure 2:**
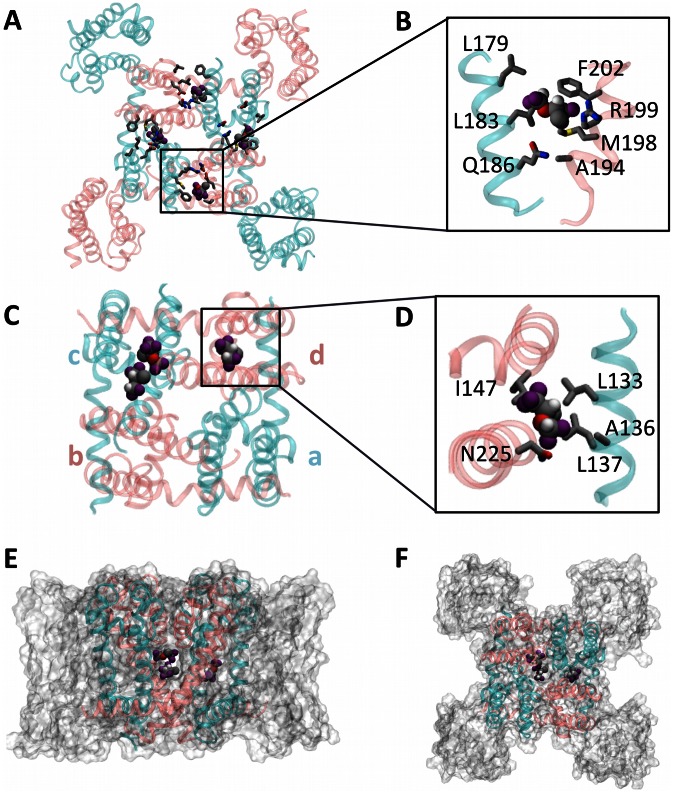
Isoflurane binding sites. Extracellular (A and B) and linker site (C and D): residues interacting with isoflurane are highlighted in the insets B and D. For clarity, alternating subunits are shown in cyan/pink and labeled a through d. (E and F) Side and top views of isoflurane molecules occupying the cavity site and fenestrations.

The extracellular site sits at the intersubunit interface between the P-loops (Figure 2, A and B). The majority of drug-sidechain interactions in this site are non-polar (E172, L179, L182, W193, A194, M198, R199, F202), however a hydrogen-bond-like interaction exists between fluorine atoms of isoflurane and Gln 186. Isoflurane enters all four equivalent cavities within a few nanoseconds and remains there for the duration of the simulation (Table S1). The estimated binding free energy (ΔG_binding_) for this site is −4.2±0.8 kcal/mol. Consistent with the notion of a tight-binding interaction, rotational and translational diffusion in this site are restricted compared to aqueous solution (Table S2). Additionally, the corresponding diffusion constants are the smallest among all sites (Table S2).

#### Linker site

The linker site is located at the “corner” formed by the N-terminus of one linker and the C-terminus of the adjacent linker (Figure 2, B and C). This site is the most dynamic and heterogeneous of the three sites, with variable occupancy and exchange of isoflurane molecules. Unlike the other sites, this site is asymmetrically occupied, with the a–d and c–d sites (Figure 2, legend) continuously occupied, while the other two equivalent sites are not occupied for any significant length of time over the course of the simulation (Table S1). Either one or two isoflurane molecules occupy the a–d site, while the c–d site is occupied by only one molecule. Also for this site, the majority of drug-protein interactions are non-polar (Leu133, Ala136 and Ile147) but a hydrogen–bonding–like interaction exists with Asn 225. The estimated ΔG_binding_ for this site is −3.7±−0.4 kcal/mol for the first isoflurane molecule and −2.6±−0.9 for the second molecule, given the first one is already bound. In contrast to the extracellular binding site, only translational diffusion is significantly slower compared to aqueous solution, suggesting that isoflurane retains a significant degree of conformational flexibility inside the pocket.

#### Pore site

**Figure 3:**
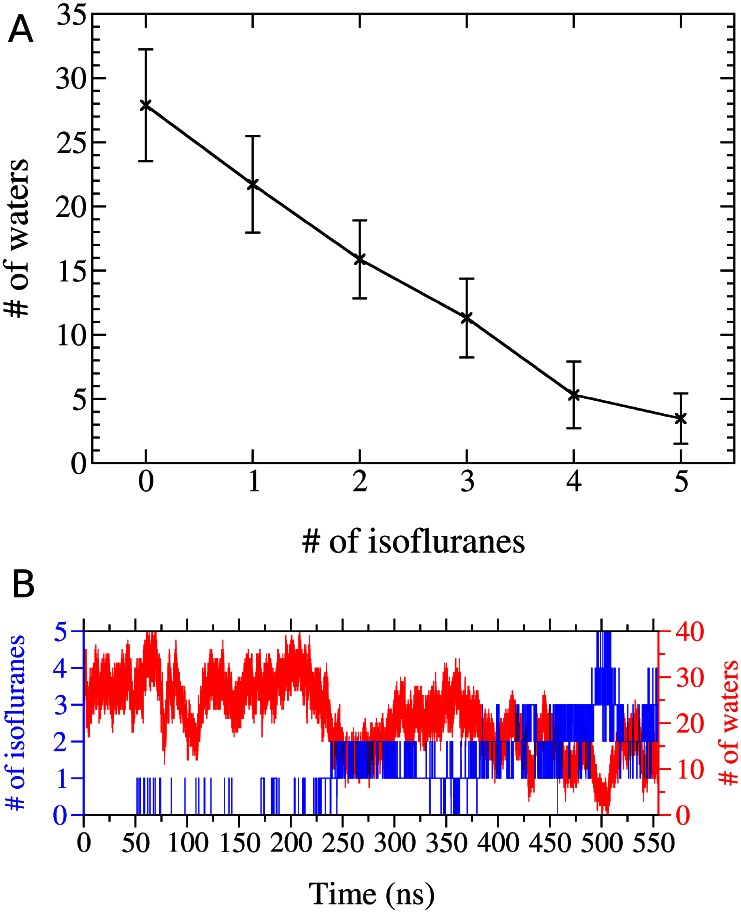
Isoflurane displacement of cavity water molecules. (A) Absolute number of water molecules (red) and isoflurane molecules (blue) within the cavity as a function of time. (B) Number of cavity waters as a function of the number of bound isoflurane molecules. Note that each isoflurane molecule displaces approximately 6 waters.

Though the activation-gate is closed, we find that the central cavity is occupied by up to five isoflurane molecules at once (Figure 2, D and E, Figure 3, A and B). All interactions in the pore are hydrophobic and isoflurane molecules associate with a number of residues. Importantly, we observed a stable interaction with residues known to play a role in local anesthetic action in mammalian sodium channels (F1764 and Y1771 in Nav1.2), which are partially conserved in NaChBac (T220 and F227) [8], [10] (Table S1). Despite the fact that we observe exchange between the central cavity and the hydrophobic section of the lipid bilayer, the cavity is almost always occupied by at least one drug molecule (Figure 3 B). Therefore we expect the affinity for this site to be comparable to those for the other sites.

Given that isoflurane molecules in the central cavity are in direct contact with water, we were surprised that diffusion translational constant is comparable to those observed in the other two binding sites.

#### Fenestrations

**Figure 4:**
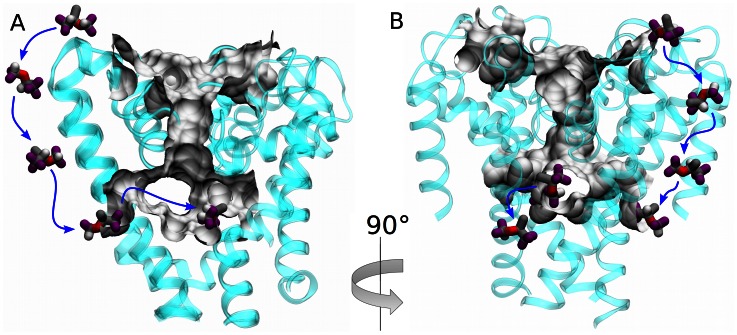
Fenestrations as the hydrophobic access pathway. A and B show two views of an isoflurane molecule diffusing in (A) and out (B) of the central cavity through two adjacent fenestrations. Cavity and fenestrations are shown as grey surfaces.

Our homology model of NaChBac preserves the fenestrations found in the NavAb crystal structure [21], [26], which provide a hydrophobic tunnel from the protein surface to the central cavity. We anticipated isoflurane occupation of hydrophobic pockets on the NaChBac protein surface, but were unsure whether isoflurane molecules would be able to fully traverse the fenestrations to displace bound waters and access the pore. In the flooding simulation, the S6 gate was closed, and there was no hydrophilic pathway for the drug molecules to the central cavity. Nevertheless, several isoflurane molecules were able to reach the pore through the fenestrations, displacing water molecules that left the central cavity through the selectivity filter. Interestingly, each isoflurane molecule is able to displace approximately six water molecules (Figure 3A). Thus, in presence of a drug molecule, two solvation shells of water cannot surround a permeant sodium ion. An intriguing hypothesis raised by this observation is that inhibition may result from desolvation of the central cavity rather than steric hindrance, therefore a correlation between molecular volume of the drug and potency should be expected for this site. We found that isoflurane can both enter and exit the pore via fenestrations (Figure 4, Movie S2) and that all four fenestrations had both exchange and occupation by isoflurane (Table S1). This observation strongly suggests that fenestrations can act as a hydrophobic access pathway to the central cavity for small hydrophobic drugs such as isoflurane. However, it is still unclear if larger hydrophobic drugs, including other general anesthetics like sevoflurane and propofol, could traverse the same fenestration pathway. Crystal structures of NavBacs have so far shown that the fenestrations are occupied by the alkyl moiety of lipid molecules. Our simulations confirm that in the absence of isoflurane, each fenestration is occupied by the alkyl moiety of a lipid molecule; but in the presence of isoflurane, the lipid tails are displaced allowing isoflurane to enter the central cavity.

## Discussion

Using an unbiased sampling of cavities within the bacterial channel NaChBac, we identified three putative binding sites for isoflurane. These binding sites are located at the extracellular side of the channel near the selectivity filter, at the linker between S4 and S5, and in the central cavity. Though the estimated binding free energies indicate high affinity, isoflurane molecules explore several orientations within each binding site. Due to the flexible nature of the Asn and Gln side-chains, isoflurane is able to form stable interactions with the protein at the extracellular and linker sites. Surprisingly, despite the fact that the pore is not accessible from the intracellular side, isoflurane molecules were able to reach the central cavity through the fenestrations. This observation suggests that the fenestrations could be the anesthetic hydrophobic pathways proposed more than three decades ago [12], [13], [24].

### Possible mechanisms of anesthetic action

All three identified sites are in regions of the protein that are predicted by mutagenesis to be critical to gating and conduction, and we hypothesize that some or all of them will play a role in inhibition of NaChBac by isoflurane. Since the time-scales involved in the molecular events relevant for gating are beyond normal MD simulation time-scales, we cannot directly probe the effect of drug interactions on protein conformation. However, based on knowledge of the mechanisms of gating, conduction, and inhibition by local anesthetics, we postulate possible mechanisms of drug action involving the binding sites suggested by our simulations. Isoflurane binding to the extracellular site positioned in the P-loops could affect the conformation of the selectivity filter, leading to inactivation through filter collapse [16],[17] and consequent reduction in peak current. Isoflurane binding to the linker site, which sits between the cuff formed by the S4–S5 linkers and the S6 bundle crossing, could affect the conformation or the coupling between the cuff and the bundle crossing to halt gating [30]–[32]. Finally, isoflurane in the pore site could simply occlude the pore through classical local anesthetic mechanisms [1], which has also been recently proposed as an inhibition mechanism of the GLIC channel by the same anesthetic [33]; this open channel block could be either purely steric or enhanced by the desolvation of the pore.

Though theories of anesthetic action focus primarily on anesthetic-protein interactions, the role of lipids cannot be discounted as partitioning into the bilayer changes lipid properties [34], [35]. For the structurally unrelated channel gramicidin the effect of halothane on channel's function has been shown to be dependent on lipid composition [36]. Channel interactions with lipids are important in regulating voltage sensing, gating and voltage-sensor-pore coupling [37]–[39] in voltage-gated ion channels. NaChBac's lipid sensitivity [40] suggests that appropriate lipid interactions regulate NaChBac function, and thus anesthetic partitioning could indirectly regulate the channel via disruption of lipid interactions. However, this simulation study is not designed to give realistic insights into lipid-anesthetic regulation for two reasons: first, because the lipid composition is not representative of a realistic bacterial or mammalian membrane and second, because the time-scale of the simulation is too short to observe lipid effects on channel structure and dynamics.

### Testable hypotheses

This simulation of isoflurane binding sites and access pathways offers a number of experimentally testable hypotheses. While simulation shows that it is thermodynamically favorable for isoflurane to occupy these sites, it is possible that isoflurane occupation may have little or no physiological function. The functional relevance of each site can be ascertained by mutating each site individually to remove key determinants of isoflurane binding and evaluating whether NaChBac retains isoflurane sensitivity through electrophysiological assays [4]. The putative hydrogen bonding partners in the extracellular and linker sites (Gln 186, Asn 225 respectively) are particularly good candidates for mutagenesis, as point mutations changing the polar character of the residue are likely to cause a large change in binding affinity at these sites. Mutation of the cavity is likely to prove difficult, as isoflurane is highly mobile and interactions are nonspecific – the cavity site may be better probed through classical use-dependence and trapping assays [1]. Most intriguingly, the relevance of the fenestrations as a drug access pathway could also be probed through mutations that occlude the fenestration pore at the extracellular side.

### Comparison to other anesthetic-channel simulations

Though no one has previously performed molecular dynamics simulation on voltage-gated sodium channels to identify anesthetic sites, numerous simulations have been done on ligand-gated ion channels (LGICs) due to the availability of the structures of bacterial homologues [41]–[43]. Simulation of anesthetic sites in LGICs, primarily based on the ELIC structure, have identified predominantly intersubunit sites, as well as a few intrasubunit sites [33], [44]–[50]. These sites are also the most common types identified experimentally [51], [52], though pore sites have also been found [45], [52]–[55]. In fact, all isoflurane sites identified in NaChBac are intersubunit sites, with the pore site also being intersubunit. Importantly, the observation that binding occurs at the intersubunit regions in both voltage- and ligand-gated channels suggests that anesthetics may impair the cooperative conformational transitions that have been shown to be crucial for the function of ion channels.

### Conclusions

Experimental verification of the aforementioned predictions will prove crucial to deepen our understanding of sodium channels modulation by anesthetics and will likely prompt further computational investigations. Indeed, despite providing a relatively accurate and detailed description of drug-channel binding events, our computational model is characterized by several limitations: (i) simulations were performed on a homology-based model of NaChBac; (ii) only one of the metastable structural conformations of the channel was probed for drug binding; (iii) lipid dependence of drug action was not addressed. Though the high degree of sequence identity with NavAb gives us confidence on the overall architecture of NaChBac, inaccuracies in the structure of the binding sites or major reorganizations of these pockets along the activation pathway can both potentially affect our results. Increased availability of experimental structures [22], [56] and a detailed characterization of the conformational transitions entailed by the activation/deactivation cycle [57] will allow us to address these issues in future computational studies, as well as being able to begin to work with eukaryotic channels [58], [59].

## Methods

### MD simulations

Simulations were initialized using the theoretical model of NaChBac in a closed conformation obtained previously [26]. This homology model was built on the basis of the X-ray crystal structure of NavAb (PDB ID: 3RVY), which was crystallized in a closed-pore conformation with all four voltage-sensing domains partially activated [57].

The model for the closed NaChBac channel, embedded in a fully hydrated lipid bilayer, was equilibrated by MD simulation. Specifically, the membrane is comprised of 1-palmytoyl-2-oleoyl-sn-glycero-3-phosphatidylcholine (POPC) lipid molecules. Isoflurane molecules were initially placed in the aqueous phase with random positions and orientation. The membrane protein complex contained a total of ∼120,000 atoms, including NaChBac, 434 lipid molecules, 25310 water molecules, 236 ions in solution and 145 isoflurane molecules. The resulting initial aqueous concentration of isoflurane is 300 mM; the equilibrium aqueous concentration 0.9 mM. Two Na^+^ ions were initially placed in the channel selectivity filter, in agreement with a previous computational study of NavAb showing double occupancy of the filter by Na^+^ ions [60]. All charged amino acids were protonated using their respective pKas and the assumption that the solution was at pH 7. MD trajectories were collected for 0.5 µs.

MD simulation used the CHARMM22-CMAP force field with torsional cross-terms for the protein and CHARMM27 for the phospholipids [61], [62]. A united-atom representation was adopted for the acyl chains of the POPC lipid molecules [63]. The water molecules were described using the TIP3P model [64], [65]. Periodic boundary conditions were employed for all of the MD simulations and the electrostatic potential was evaluated using the particle-mesh Ewald method [66]. The lengths of all bonds containing hydrogen were constrained with the SHAKE/RATTLE algorithm [67]. The system was maintained at a temperature of 300 K and pressure of 1 atm using the Langevin thermostat and barostat methods as implemented in the MD code NAMD2.8 [68] (Figure S1). The rRESPA multiple time step method was employed, with a high frequency timestep of 2.0 fs and a low frequency time step of 4.0 fs.

This computational setup has several limitations. First, the time-scales achievable by our MD simulation are too short to observe channel gating. Second, the small size of the lipid bilayer in the simulation box results in an excessive drug concentration in the bilayer. The final limitation stems from the use of a oversimplified POPC lipid bilayer. Bacterial sodium channel function is strongly lipid dependent [40]. However, NaChBac is functional in liposomes containing POPC and in mammalian cells, making POPC a reasonable choice for NaChBac simulations.

### Cluster analysis

To identify binding sites, we analyzed equilibrated configurations of the system and seeking regions characterized by high density of isoflurane. We first computed the center of mass (COM) of each isoflurane molecule in the MD trajectory frame. We then performed a cluster analysis on the resulting set of COM positions using the geometric distance between each pair of positions to build a proximity matrix. Partitioning of the set (clustering) was obtained using the Jarvis-Patrick algorithm [27] with a nearest-neighbor list of 8 and shared-neighbor threshold of 3. We then ranked the clusters according to their average density. Here, we treated each COM position as an isotropic Gaussian density of width 1 Å, and summed over all the COM positions belonging to a given cluster. Integration of the density for each cluster was performed on a 3-dimensional grid with bin dimensions of 0.5×0.5×0.5 Å^3^. The top three ranked clusters, comprising 15% of the total number of configurations, are discussed as putative binding regions.

### Free energy calculations

Free energy calculations were performed using the free energy perturbation (FEP) method. The binding free energies are calculated using the following scheme: DG_bind_ = DG_gas–prot_−(DG_solv_+DG_rstr_). Here, DG_bind_ is the free energy of binding isoflurane to NaChBac, DG_vac->prot_ is the free energy of transferring an isoflurane from the gas phase to the binding site, DG_solv_ is the isoflurane solvation free energy, and DG_rstr_ is a measure of the entropy cost associated with the reduction in volume from a 1 M solution (V_1M_) to the volume available at the binding site (V_rstr_), i.e., DG_rstr_ = RT ln(V_rstr_/V_1M_). For all reported binding energies, V_1M_ is given by the volume associated with the flat-bottom spherical restraint applied to keep the isoflurane in the binding site during the interaction decoupling. Calculations were performed in NAMD 2.8 by varying the coupling parameter in steps of 0.025 at the ends and 0.05 in the middle.

This approach has been successfully applied to binding of anesthetics to proteins including the binding of R- and S- isoflurane enantiomers to apoferritin [69] as well as the binding of isoflurane and propofol to the GLIC bacterial ion channel [33]. The estimated binding free energy for isoflurane was assuming that the only two relevant thermodynamic states are protein-bound isoflurane aqueous isoflurane. Therefore the water-lipid partitioning is not considered. However, since the affinity of isoflurane for a hydrophobic phase is significantly lower than our estimated affinities (ΔG of transfer from water to dodecane is 3.0 kcal/mol), we expect the lipid partitioning to have a marginal effect on the apparent K_d_.

## Supporting Information

Figure S1 (A) Left: displacement along the bilayer normal of the centers of mass of the isoflurane molecules as a function of time. Different colors are used to show the instantaneous positions of different isoflurane molecules. Right: density profile along the bilayer normal calculated for the centers of mass of all the isoflurane molecules averaged over the first 100 ns (black) and the subsequent 450 ns (red). Note that in the latter the values of the density outside the bilayer region are negligible. (B) Plot showing number of isoflurane molecules in aqueous phase over time. Inset show magnified scale for 100–550 ns, after isoflurane is fully partitioned. (C) Probability distribution function of the number of isoflurane molecules computed over the last 100 ns of simulation. The average number of isoflurane in water over this period is 0.4.
(TIFF)

Figure S2 Root mean square deviation (RMSD) of the backbone atoms from the initial structure employed in the MD simulation plotted as a function of time for the flooding simulation. The RMSD is shown separately for different regions of the channel: entire channel (black), voltage-sensor domains (blue), pore domain (red), and selectivity filter (green).
(TIFF)

Movie S1 Isoflurane flooding. The NaChBac pore domain (blue ribbons) sits in the bilayer (headgroups shown as grey spheres) while isoflurane (red/green molecules) begins in the aqueous compartment above and below the bilayer and partitions first into the bilayer and then into the protein structure. Voltage sensing domains, water molecules and lipid alkyl tails are not shown in this movie but are present in the simulation (Figure 1A).
(MPG)

Movie S2 Representative isoflurane trajectory through fenestration to cavity site. Bottom view of NaChBac structure (grey ribbons) surrounded by lipid molecules (stick representations). Isoflurane (blue, space-filling representation) enters from the lipid phase into the cavity through a fenestration.
(MPG)

Table S1 Proximity of isoflurane to residues within each site. The table reports the relative frequencies (as a percentage of frames) of contacts between isoflurane and the residues lining each binding site (a contact is detected if the distance between any atom of the isoflurane molecule and any atom of the given residue is smaller than 5 Å). Residues are classified as non-interacting (red), possibly interacting (orange), and likely interacting (green) depending on the probability of being engaged in a contact.
(DOCX)

Table S2 Dynamics of Isoflurane in binding sites. Diffusion coefficients and rotational relaxation times of isoflurane computed, at each binding site, by averaging over a trajectory of approximately 500 ns. The water self-diffusion coefficient and the rotational relaxation time for the TIP3P model is reported for comparison.
(DOCX)

## References

[1] Hille B (2001) Ion channels of excitable membranes. Sunderland, Mass.: Sinauer.

[2] Ratnakumari L, Hemmings HC (1998) Inhibition of presynaptic sodium channels by halothane. Anesthesiology 88: 1043–1054. 9579514 10.1097/00000542-199804000-00025

[3] Ouyang W, Wang G, Hemmings HC (2003) Isoflurane and propofol inhibit voltage-gated sodium channels in isolated rat neurohypophysial nerve terminals. Mol Pharmacol 64: 373–381. 12869642 10.1124/mol.64.2.373

[4] Ouyang W, Jih T-Y, Zhang T-T, Correa AM, Hemmings HC (2007) Isoflurane inhibits NaChBac, a prokaryotic voltage-gated sodium channel. J Pharmacol Exp Ther 322: 1076–1083. 17569823 10.1124/jpet.107.122929

[5] Ouyang W, Herold KF, Hemmings HC (2009) Comparative effects of halogenated inhaled anesthetics on voltage-gated Na+ channel function. Anesthesiology 110: 582–590. 19225394 10.1097/ALN.0b013e318197941e PMC2699670

[6] Herold KF, Nau C, Ouyang W, Hemmings HC (2009) Isoflurane inhibits the tetrodotoxin-resistant voltage-gated sodium channel Nav1.8. Anesthesiology 111: 591–599. 19672182 10.1097/ALN.0b013e3181af64d4 PMC2756082

[7] Herold KF, Hemmings HC (2012) Sodium channels as targets for volatile anesthetics. Front Pharmacol 3: 50. 22479247 10.3389/fphar.2012.00050 PMC3316150

[8] Lee S, Goodchild SJ, Ahern CA (2012) Local anesthetic inhibition of a bacterial sodium channel. J Gen Physiol 139: 507–516. 22641643 10.1085/jgp.201210779 PMC3362524

[9] Courtney KR (1975) Mechanism of frequency-dependent inhibition of sodium currents in frog myelinated nerve by the lidocaine derivative GEA. J Pharmacol Exp Ther 195: 225–236. 1081138

[10] Ragsdale DS, McPhee JC, Scheuer T, Catterall WA (1994) Molecular determinants of state-dependent block of Na+ channels by local anesthetics. Science 265: 1724–1728. 8085162 10.1126/science.8085162

[11] Hille B, Courtney K, Dum R (1975) Rate and site of local anesthetics in myelinated nerve fibers. In: Fink BR, editor. Molecular Mechanisms of Anesthesia. New York: Raven Press.

[12] Hille B (1977) Local anesthetics: hydrophilic and hydrophobic pathways for the drug-receptor reaction. J Gen Physiol 69: 497–515. 300786 10.1085/jgp.69.4.497 PMC2215053

[13] Schwarz W, Palade PT, Hille B (1977) Local anesthetics. Effect of pH on use-dependent block of sodium channels in frog muscle. Biophys J 20: 343–368. 21711 10.1016/S0006-3495(77)85554-9 PMC1473363

[14] Ren D, Navarro B, Xu H, Yue L, Shi Q, Clapham DE (2001) A prokaryotic voltage-gated sodium channel. Science 294: 2372–2375. 11743207 10.1126/science.1065635

[15] Koishi R, Xu H, Ren D, Navarro B, Spiller BW, et al (2004) A superfamily of voltage-gated sodium channels in bacteria. J Biol Chem 279: 9532–9538. 14665618 10.1074/jbc.M313100200

[16] Pavlov E, Bladen C, Winkfein R, Diao C, Dhaliwal P, French RJ (2005) The pore, not cytoplasmic domains, underlies inactivation in a prokaryotic sodium channel. Biophys J 89: 232–242. 15849254 10.1529/biophysj.104.056994 PMC1366521

[17] Irie K, Kitagawa K, Nagura H, Imai T, Shimomura T, Fujiyoshi Y (2010) Comparative study of the gating motif and C-type inactivation in prokaryotic voltage-gated sodium channels. J Biol Chem 285: 3685–3694. 19959480 10.1074/jbc.M109.057455 PMC2823509

[18] Doyle DA, Cabral JM, Pfuetzner RA, Kuo A, Gulbis JM, et al (1998) The structure of the potassium channel: molecular basis of K+ conduction and selectivity. Science 280: 69. 9525859 10.1126/science.280.5360.69

[19] Townsend C, Horn R (1999) Interaction between the pore and a fast gate of the cardiac sodium channel. J Gen Physiol 113: 321–332. 9925827 10.1085/jgp.113.2.321 PMC2223368

[20] Kuo CC, Liao SY (2000) Facilitation of recovery from inactivation by external Na+ and location of the activation gate in neuronal Na+ channels. J Neurosci 20: 5639–5646. 10908601 10.1523/JNEUROSCI.20-15-05639.2000 PMC6772556

[21] Payandeh J, Scheuer T, Zheng N, Catterall WA (2011) The crystal structure of a voltage-gated sodium channel. Nature 475: 353–358. 21743477 10.1038/nature10238 PMC3266868

[22] Zhang X, Ren W, DeCaen P, Yan C, Tao X, et al (2012) Crystal structure of an orthologue of the NaChBac voltage-gated sodium channel. Nature 486: 130–134. 22678295 10.1038/nature11054 PMC3979295

[23] Payandeh J, Gamal El-Din TM, Scheuer T, Zheng N, Catterall WA (2012) Crystal structure of a voltage-gated sodium channel in two potentially inactivated states. Nature 486: 135–139. 22678296 10.1038/nature11077 PMC3552482

[24] Hondeghem LM, Katzung BG (1977) Time- and voltage-dependent interactions of antiarrhythmic drugs with cardiac sodium channels. Biochim Biophys Acta 472: 373–398. 334262 10.1016/0304-4157(77)90003-x

[25] Vemparala S, Domene C, Klein ML (2008) Interaction of anesthetics with open and closed conformations of a potassium channel studied via molecular dynamics and normal mode analysis. Biophys J 94: 4260–4269. 18310250 10.1529/biophysj.107.119958 PMC2480689

[26] Barber AF, Carnevale V, Raju SG, Amaral C, Treptow W, Klein ML (2012) Hinge-bending motions in the pore domain of a bacterial voltage-gated sodium channel. Biochim Biophys Acta 1818: 2120–2125. 22579978 10.1016/j.bbamem.2012.05.002 PMC3378804

[27] Jarvis RA, Patrick EA (1973) Clustering using a similarity measure based on shared near neighbors. Computers, IEEE Transactions on 100: 1025–1034.

[28] Pohorille A, Jarzynski C, Chipot C (2010) Good practices in free-energy calculations. J Phys Chem B 114: 10235–10253. 20701361 10.1021/jp102971x

[29] Franks NP, Lieb WR (1996) Temperature dependence of the potency of volatile general anesthetics: implications for in vitro experiments. Anesthesiology 84: 716–720. 8659800 10.1097/00000542-199603000-00027

[30] Bezanilla F (2000) The voltage sensor in voltage-dependent ion channels. Physiol Rev 80: 555. 10747201 10.1152/physrev.2000.80.2.555

[31] Yifrach O, MacKinnon R (2002) Energetics of pore opening in a voltage-gated K(+) channel. Cell 111: 231–239. 12408867 10.1016/s0092-8674(02)01013-9

[32] Long SB, Campbell EB, Mackinnon R (2005) Crystal structure of a mammalian voltage-dependent Shaker family K+ channel. Science 309: 897–903. 16002581 10.1126/science.1116269

[33] LeBard DN, Hénin J, Eckenhoff RG, Klein ML, Brannigan G (2012) General anesthetics predicted to block the GLIC pore with micromolar affinity. PLoS Comput Biol 8: e1002532. 22693438 10.1371/journal.pcbi.1002532 PMC3364936

[34] Weinrich M, Nanda H, Worcester DL, Majkrzak CF, Maranville BB, Bezrukov SM (2012) Halothane Changes the Domain Structure of a Binary Lipid Membrane. Langmuir 28: 4723–4728. 22352350 10.1021/la204317k PMC3302933

[35] Turkyilmaz S, Almeida PF, Regen SL (2011) Effects of isoflurane, halothane, and chloroform on the interactions and lateral organization of lipids in the liquid-ordered phase. Langmuir 27: 14380–14385. 21995557 10.1021/la2035278 PMC3226895

[36] Weinrich M, Rostovtseva TK, Bezrukov SM (2009) Lipid-dependent effects of halothane on gramicidin channel kinetics: a new role for lipid packing stress. Biochemistry 48: 5501–5503. 19405539 10.1021/bi900494y PMC3419375

[37] Milescu M, Bosmans F, Lee S, Alabi AA, Kim JI, Swartz KJ (2009) Interactions between lipids and voltage sensor paddles detected with tarantula toxins. Nat Struct Mol Biol 16: 1080–1085. 19783984 10.1038/nsmb.1679 PMC2782670

[38] Mokrab Y, Sansom MSP (2011) Interaction of diverse voltage sensor homologs with lipid bilayers revealed by self-assembly simulations. Biophys J 100: 875–884. 21320431 10.1016/j.bpj.2010.11.049 PMC3037555

[39] Morris CE, Juranka PF, Joós B (2012) Perturbed voltage-gated channel activity in perturbed bilayers: Implications for ectopic arrhythmias arising from damaged membrane. Prog Biophys Mol Biol 110: 245–256. 22846437 10.1016/j.pbiomolbio.2012.07.003

[40] D'Avanzo N, McCusker EC, Powl AM, Miles AJ, Nichols CG, et al (2013) Differential Lipid Dependence of Function of Bacterial Sodium Channel Homologues. Biophys J 104: 14a. 10.1371/journal.pone.0061216 PMC3620320 23579615

[41] Hilf RJC, Dutzler R (2008) X-ray structure of a prokaryotic pentameric ligand-gated ion channel. Nature 452: 375–379. 18322461 10.1038/nature06717

[42] Bocquet N, Nury H, Baaden M, Le Poupon C, Changeux J-P, et al (2009) X-ray structure of a pentameric ligand-gated ion channel in an apparently open conformation. Nature 457: 111–114. 18987633 10.1038/nature07462

[43] Hilf RJC, Dutzler R (2009) Structure of a potentially open state of a proton-activated pentameric ligand-gated ion channel. Nature 457: 115–118. 18987630 10.1038/nature07461

[44] Chen Q, Cheng MH, Xu Y, Tang P (2010) Anesthetic binding in a pentameric ligand-gated ion channel: GLIC. Biophys J 99: 1801–1809. 20858424 10.1016/j.bpj.2010.07.023 PMC2941008

[45] Brannigan G, LeBard DN, Hénin J, Eckenhoff RG, Klein ML (2010) Multiple binding sites for the general anesthetic isoflurane identified in the nicotinic acetylcholine receptor transmembrane domain. Proc Natl Acad Sci USA 107: 14122–14127. 20660787 10.1073/pnas.1008534107 PMC2922517

[46] Murail S, Wallner B, Trudell JR, Bertaccini E, Lindahl E (2011) Microsecond Simulations Indicate that Ethanol Binds between Subunits and Could Stabilize an Open-State Model of a Glycine Receptor. Biophys J 100: 1642–1650. 21463577 10.1016/j.bpj.2011.02.032 PMC3072665

[47] Howard RJ, Murail S, Ondricek KE, Corringer P-J, Lindahl E, et al (2011) Structural basis for alcohol modulation of a pentameric ligand-gated ion channel. Proc Natl Acad Sci USA 108: 12149–12154. 21730162 10.1073/pnas.1104480108 PMC3141919

[48] Willenbring D, Liu LT, Mowrey D, Xu Y, Tang P (2011) Isoflurane Alters the Structure and Dynamics of GLIC. Biophys J 101: 1905–1912. 22004744 10.1016/j.bpj.2011.09.026 PMC3192980

[49] Murail S, Howard RJ, Broemstrup T, Bertaccini EJ, Harris RA, et al (2012) Molecular mechanism for the dual alcohol modulation of Cys-loop receptors. PLoS Comput Biol 8: e1002710. 23055913 10.1371/journal.pcbi.1002710 PMC3464191

[50] Pan J, Chen Q, Willenbring D, Mowrey D, Kong X-P, et al (2012) Structure of the pentameric ligand-gated ion channel GLIC bound with anesthetic ketamine. Structure 20: 1463–1469. 22958642 10.1016/j.str.2012.08.009 PMC3446250

[51] Krasowski MD, Harrison NL (1999) General anaesthetic actions on ligand-gated ion channels. Cell Mol Life Sci 55: 1278–1303. 10487207 10.1007/s000180050371 PMC2854026

[52] Forman SA (2011) Clinical and molecular pharmacology of etomidate. Anesthesiology 114: 695–707. 21263301 10.1097/ALN.0b013e3181ff72b5 PMC3108152

[53] Forman SA, Miller KW, Yellen G (1995) A discrete site for general anesthetics on a postsynaptic receptor. Mol Pharmacol 48: 574–581. 7476881

[54] Wenningmann I, Barann M, Vidal AM, Dilger JP (2001) The Effects of isoflurane on acetylcholine receptor channels: 3. Effects of conservative polar-to-nonpolar mutations within the channel pore. Mol Pharmacol 60: 584–594. 11502891

[55] Nirthanan S, Garcia G, Chiara DC, Husain SS, Cohen JB (2008) Identification of binding sites in the nicotinic acetylcholine receptor for TDBzl-etomidate, a photoreactive positive allosteric effector. J Biol Chem 283: 22051–22062. 18524766 10.1074/jbc.M801332200 PMC2494931

[56] McCusker EC, Bagnéris C, Naylor CE, Cole AR, D'Avanzo N, et al (2012) Structure of a bacterial voltage-gated sodium channel pore reveals mechanisms of opening and closing. Nat Commun 3: 1102. 23033078 10.1038/ncomms2077 PMC3493636

[57] Amaral C, Carnevale V, Klein ML, Treptow W (2012) Exploring conformational states of the bacterial voltage-gated sodium channel NavAb via molecular dynamics simulations. Proc Natl Acad Sci USA 10.1073/pnas.1218087109 PMC3535635 23150565

[58] Bruhova I, Tikhonov DB, Zhorov BS (2008) Access and binding of local anesthetics in the closed sodium channel. Mol Pharmacol 74: 1033–1045. 18653802 10.1124/mol.108.049759

[59] Tikhonov DB, Zhorov BS (2012) Architecture and pore block of eukaryotic voltage-gated sodium channels in view of NavAb bacterial sodium channel structure. Mol Pharmacol 82: 97–104. 22505150 10.1124/mol.112.078212

[60] Carnevale V, Treptow W, Klein ML (2011) Sodium Ion Binding Sites and Hydration in the Lumen of a Bacterial Ion Channel from Molecular Dynamics Simulations. J Phys Chem Lett 2: 2504–2508.

[61] MacKerell AD, Banavali N, Foloppe N (2000) Development and current status of the CHARMM force field for nucleic acids. Biopolymers 56: 257–265. 11754339 10.1002/1097-0282(2000)56:4<257::AID-BIP10029>3.0.CO;2-W

[62] Mackerell AD, Feig M, Brooks CL (2004) Extending the treatment of backbone energetics in protein force fields: limitations of gas-phase quantum mechanics in reproducing protein conformational distributions in molecular dynamics simulations. J Comput Chem 25: 1400–1415. 15185334 10.1002/jcc.20065

[63] Hénin J, Shinoda W, Klein ML (2008) United-atom acyl chains for CHARMM phospholipids. J Phys Chem B 112: 7008–7015. 18481889 10.1021/jp800687p PMC8451178

[64] Jorgensen WL, Chandrasekhar J, Madura JD, Impey RW, Klein ML (1983) Comparison of simple potential functions for simulating liquid water. J Chem Phys 79: 926.

[65] Darve E, Rodríguez-Gómez D, Pohorille A (2008) Adaptive biasing force method for scalar and vector free energy calculations. J Chem Phys 128: 144120. 18412436 10.1063/1.2829861

[66] Essmann U, Perera L, Berkowitz ML, Darden T, Lee H, Pedersen LG (1995) A smooth particle mesh Ewald method. J Chem Phys 103: 8577.

[67] Ryckaert JP, Ciccotti G, Berendsen HJC (1977) Numerical integration of the cartesian equations of motion of a system with constraints: molecular dynamics of *n*-alkanes. J Comput Phys 23: 327–341.

[68] Phillips JC, Braun R, Wang W, Gumbart J, Tajkhorshid E, et al (2005) Scalable molecular dynamics with NAMD. J Comput Chem 26: 1781–1802. 16222654 10.1002/jcc.20289 PMC2486339

[69] Hénin J, Brannigan G, Dailey WP, Eckenhoff R, Klein ML (2010) An atomistic model for simulations of the general anesthetic isoflurane. J Phys Chem B 114: 604–612. 19924847 10.1021/jp9088035

