# Supplementary figures and images for: Exploring Volatile General Anesthetic Binding to a Closed Membrane-Bound Bacterial Voltage-Gated Sodium Channel via Computation
Source: PLoS Comput Biol. 2013 Jun 13;9(6):e1003090. doi: 10.1371/journal.pcbi.1003090 (PMC3681623; doi:10.1371/journal.pcbi.1003090)

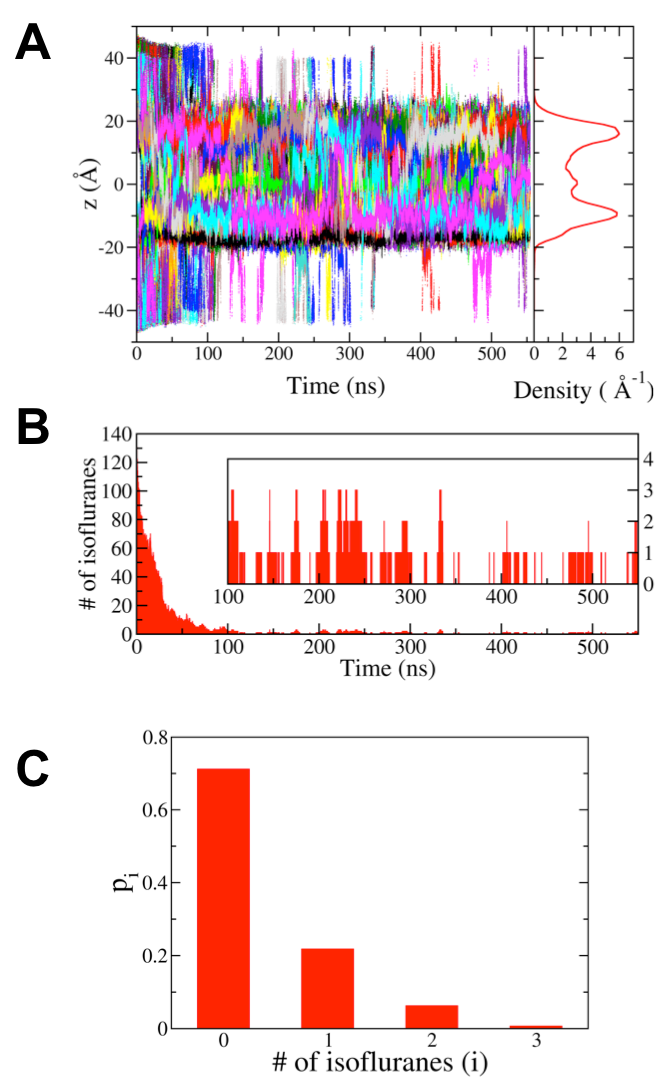

Supplement: Figure S1 — (A) Left: displacement along the bilayer normal of the centers of mass of the isoflurane molecules as a function of time. Different colors are used to show the instantaneous positions of different isoflurane molecules. Right: density profile along the bilayer normal calculated for the centers of mass of all the isoflurane molecules averaged over the first 100 ns (black) and the subsequent 450 ns (red). Note that in the latter the values of the density outside the bilayer region are negligible. (B) Plot showing number of isoflurane molecules in aqueous phase over time. Inset show magnified scale for 100–550 ns, after isoflurane is fully partitioned. (C) Probability distribution function of the number of isoflurane molecules computed over the last 100 ns of simulation. The average number of isoflurane in water over this period is 0.4. (TIFF) [file pcbi.1003090.s001.tiff]

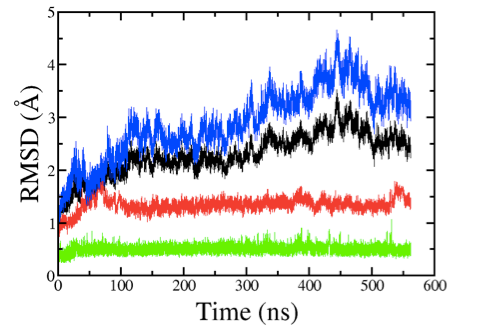

Supplement: Figure S2 — Root mean square deviation (RMSD) of the backbone atoms from the initial structure employed in the MD simulation plotted as a function of time for the flooding simulation. The RMSD is shown separately for different regions of the channel: entire channel (black), voltage-sensor domains (blue), pore domain (red), and selectivity filter (green). (TIFF) [file pcbi.1003090.s002.tiff]
