# Supplementary material for: Exploring Volatile General Anesthetic Binding to a Closed Membrane-Bound Bacterial Voltage-Gated Sodium Channel via Computation
Source: PLoS Comput Biol. 2013 Jun 13;9(6):e1003090. doi: 10.1371/journal.pcbi.1003090 (PMC3681623; doi:10.1371/journal.pcbi.1003090)
Supplement: Table S1 — Proximity of isoflurane to residues within each site. The table reports the relative frequencies (as a percentage of frames) of contacts between isoflurane and the residues lining each binding site (a contact is detected if the distance between any atom of the isoflurane molecule and any atom of the given residue is smaller than 5 Å). Residues are classified as non-interacting (red), possibly interacting (orange), and likely interacting (green) depending on the probability of being engaged in a contact. (DOCX) [file pcbi.1003090.s005.docx]

| **Table S1**: Proximity of isoflurane to residues within each site. | | | | | | | |  |  |  |
| --- | --- | --- | --- | --- | --- | --- | --- | --- | --- | --- |
|  |  |  |  |  |  |  |  |  |  |  |
| **Extracellular Site** | | |  |  |  | **Cavity Site** | |  |  |  |
|  |  |  |  |  |  |  |  |  |  |  |
| **Residue** | **a** | **b** | **c** | **d** |  | **Residue** | **a** | **b** | **c** | **d** |
| **E172** | 95 | 82 | 82 | 86 |  | **T220** | 97 | 93 | 77 | 82 |
| **L179** | 98 | 97 | 84 | 87 |  | **F227** | 79 | 73 | 84 | 71 |
| **L182** | 100 | 100 | 100 | 93 |  |  |  |  |  |  |
| **Q186** | 100 | 100 | 100 | 92 |  | **Fenestration Site** | |  |  |  |
| **W193** | 20 | 35 | 36 | 33 |  |  |  |  |  |  |
| **A194** | 92 | 100 | 100 | 100 |  | **Residue** | **a** | **b** | **c** | **d** |
| **M198** | 92 | 100 | 100 | 100 |  | **F185** | 55 | 17 | 55 | 84 |
| **R199** | 94 | 99 | 100 | 94 |  | **V188** | 61 | 35 | 55 | 90 |
| **F202** | 94 | 100 | 100 | 100 |  | **T189** | 65 | 40 | 56 | 91 |
|  |  |  |  |  |  | **L190** | 75 | 74 | 60 | 67 |
| **Linker Site** | |  |  |  |  | **L217** | 92 | 87 | 31 | 88 |
|  |  |  |  |  |  | **T220** | 54 | 62 | 46 | 77 |
| **Residue** | **a** | **b** | **c** | **d** |  | **I223** | 87 | 78 | 85 | 98 |
| **L133** | 1 | 0 | 89 | 100 |  | **F224** | 100 | 98 | 93 | 94 |
| **A136** | 0 | 0 | 73 | 100 |  | **L226** | 72 | 4 | 43 | 91 |
| **L137** | 11 | 0 | 87 | 99 |  |  |  |  |  |  |
| **I147** | 100 | 0 | 5 | 88 |  | **Key:** | <10% occupancy | | |  |
| **N225** | 100 | 0 | 0 | 90 |  |  | 40-80% occupancy | | |  |
| **N234** | 1 | 0 | 89 | 93 |  |  | > 80% occupancy | | |  |
|  |  |  |  |  |  |  |  |  |  |  |
| Numbers shown here are the percent of frames in which isoflurane was within 5A of each residue listed, color coding sorts residues by non-interacting (red), possibly interacting (orange) and likely-interacting (green) based on persistence of isoflurane proximity (see key). To estimate the persistence of isoflurane proximity to residues that make up each site, we selected residues that were within 5A of the protein during *any* frame and then asked whether they were within 5A for *each* frame over the last 250 ns of trajectory. This was done separately for each subunit (a-d) to account for asymmetry. | | | | | | | | | | |
