# Supplementary material for: Exploring Volatile General Anesthetic Binding to a Closed Membrane-Bound Bacterial Voltage-Gated Sodium Channel via Computation
Source: PLoS Comput Biol. 2013 Jun 13;9(6):e1003090. doi: 10.1371/journal.pcbi.1003090 (PMC3681623; doi:10.1371/journal.pcbi.1003090)
Supplement: Table S2 — Dynamics of Isoflurane in binding sites. Diffusion coefficients and rotational relaxation times of isoflurane computed, at each binding site, by averaging over a trajectory of approximately 500 ns. The water self-diffusion coefficient and the rotational relaxation time for the TIP3P model is reported for comparison. (DOCX) [file pcbi.1003090.s006.docx]

| **Table S2:** Dynamics of Isoflurane in binding sites | | |
| --- | --- | --- |
| **Site** | **Translational diffusion coefficient (Å^2^/s)** | **Rotational relaxation time (ns)** |
| Water | 100 | 0.1 |
| Extracellular | 2 | 5.3 |
| Linker | 2 | 1 |
| Cavity | 3 | 0.1 |
| Diffusion coefficients and rotational relaxation times of isoflurane computed, at each binding site, by averaging over a trajectory of approximately 500 ns. The water self-diffusion coefficient and the rotational relaxation time for the TIP3P model is reported for comparison. | | |
